# Professor Robert Koch

**Published:** 1910-06-04

**Authors:** 


Professor Robert Koch, the bacteriologist, died on the night of May 28 at Baden-Baden, whither he had gone for change of air after an attack of heart trouble. He was 61 years of age, and was a Nobel prizeman, and Professor Extraordinary with the title of Excellency, besides the recipient of many German and foreign decorations. His work in connection with tuberculosis, anthrax, sleeping sickness, bubonic plague, and rhinoscleroma is well known. Among the many messages received by Frau Koch is one from the Emperor William, who says that Professor Koch's death removes "the greatest German doctor of our time." The body will be taken to Hamburg and cremated.

